# Ethnomedicinal Uses, Phytochemistry and Pharmacological Properties of *Suregada* Genus: A Review

**DOI:** 10.3390/ph16101390

**Published:** 2023-09-30

**Authors:** Mandisa Mangisa, Douglas Kemboi, Gerda Fouche, Rudzani Nthambeleni, Moses Kiprotich Langat, Clemence Tarirai, Martin Cheek, Odwa Gonyela, Vuyelwa Jacqueline Tembu

**Affiliations:** 1 Department of Chemistry, Tshwane University of Technology, Private Bag X680, Pretoria 0001, South Africa; mangisam@tut.ac.za (M.M.); gonyelao@tut.ac.za (O.G.); tembuvj@tut.ac.za (V.J.T.); 2 Department of Chemistry, University of Kabianga, Kericho 2030, Kenya; 3 Department of Chemistry, University of Pretoria, Private Bag X20, Pretoria 0001, South Africa; gerda.fouche@up.ac.za; 4 Biosciences, Council for Scientific and Industrial Research, Pretoria 0001, South Africa; nthambelenirudzani@gmail.com; 5 Science Department, Royal Botanic Gardens, Kew TW9 3DS, UK; m.langat@kew.org (M.K.L.); m.cheek@kew.org (M.C.); 6 Department of Pharmaceutical Sciences, Tshwane University of Technology, Private Bag X680, Pretoria 0001, South Africa; tariraic@tut.ac.za

**Keywords:** Euphorbiaceae, *Suregada*, phytochemistry, ethnomedicine, pharmacological uses

## Abstract

Plants of the *Suregada* Roxb. ex Rottler (formerly *Gelonium* Roxb. ex Willd) are utilized to treat various ailments, namely, hepatic, gum diseases, pyrexia, eczema, and venereal diseases. This review links the reported compounds to ethnomedicinal uses through pharmacological activities. The compounds possess anticancer, anti-allergic, antibacterial, anti-inflammatory, antioxidant, and anti-HIV properties. From the previous reports, 32 known species of the *Suregada* genus have been investigated morphologically, and nine were investigated for their phytochemistry and pharmacology. Phytochemistry, ethnomedicinal, and pharmacological uses of the other 23 *Suregada* species are not known and/or not reported. In this review, abietane diterpenoids are the main compounds expressed by the *Suregada*, accounting for 71 of the 114 reported compounds. Ten triterpenoids and sterols, one aliphatic, two lignans, five flavonoids, and twenty-one nitrogen-containing compounds have been reported from the genus.

## 1. Introduction

*Suregada* Roxb. ex Rottler (formerly *Gelonium* Roxb. ex Willd.) species are shrubs or small trees occurring in the forest, deciduous forest, or thicket with 32 accepted species, extending from Africa and Madagascar through India to southern New Guinea, China, Philippines, and northern Australia [1].

Based on morphology, *Suregada* was placed in Euphorbiaceae-Crotonoideae in the tribe Gelonieae with the monotypic *Cladogelonium* Leandri of Madagascar by Radcliffe-Smith in genera *Euphorbiacearum* [2,3]. Wurdack et al. stated that the relationship between these two genera was endorsed in a molecular phylogenetic study and was sister to the main clade of Crotonoid genera [4].

The genus *Suregada* is highly distinctive and cannot be confused with any other genus of tree or shrub in Sub-Saharan Africa. The combination of translucent gland dots in the leaves, leaf-opposed fasciculate or glomerulate inflorescences, and the node encircled by stipules (or stipular scars) separates it not only from all other genera in Euphorbiaceae but from all other genera with alternate simple leaves [2,3]. *Suregada* is best recognized by its leaf-opposed inflorescences; the whole plant is usually drying bright green and often has characteristic pustules within the leaf areoles [3].

The African species were last reviewed by Léonard when publishing the most recent African addition to the genus, S. *croizatiana* J. Léonard, a forest shrub from the Democratic Republic of Congo (DRC) [5]. The eight African species include *Suregada africana* Kuntze, and *S. lithoxyla* (Pax & K.Hoffm.) Croizat, *S. zanzibariensis* Baill, *S. procera* (Prain) Croizat, *S. gossweileri* (S.Moore) Croizat, *S. ivorense* (Aubrév. & Pellegr.) J. Léonard, *S. croizatiana* J. Léonard and *S. occidentale* (Hoyle) Croizat. *Suregada africana* is a shrub or tree that grows up to 3 m. It was the first described of the African species, and is southernmost in distribution, extending along the coast in the thicket and dry woodland from KwaZulu-Natal and Eastern Cape, through Eswatini to southern Mozambique [6]. *Suregada zanzibariensis* is sympatric with the former species in KwaZulu-Natal but reaches 10 m tall and extends in a coastal thicket up the coast of East Africa through Tanzania, Mozambique, and Kenya to southern Somalia, west to Angola, and east to Madagascar. *Suregada procera* is a forest shrub or small tree species, 3–8 m tall that also extends from KwaZulu-Natal northwards, in high altitude forests through Mozambique, Zimbabwe, Uganda, Zambia, Malawi, Tanzania, Kenya, eastern Democratic Republic of Congo (DRC) to South Sudan and Ethiopia [6]. *Suregada lithoxyla* is a forest shrub native to Tanzania, and *S. gossweileri* is a forest shrub of the Maiombe Mountains of Angolan Cabinda and DRC. The two West African species are *Suregada ivorense*, a tall forest tree endemic to the Ivory Coast, and *S. occidentale* (Hoyle) Croizat, a 2–3 m tall forest shrub of the Ivory Coast to western Cameroon [5,7]. Of the 32 species, pharmacological and phytochemical reports are available for only *S. aequoreum* Hance., *S. adenophora* Baill., and *S. angustifolia* Baill. *S. boiviniana* Baill, *S. gaultheriifolia* Radcl.-Sm, *S. glomerulata* (Blume) Baill., *S. lanceolata* (Willd.) Kuntze, *S. multiflora* (A. Juss.) Baill., and *S. zanzibariensis* Baill.

The medicinal application of *Suregada* species has been attributed to the presence of diverse chemical constituents such as diterpenoids, triterpenoids, flavonoids, etc. In particular, the diterpenoids about 73% isolated from *Suregada* species constitute the major chemical composition and exist as a diversity of different core skeletal frameworks. Diterpenoids, their chemical structure and biosynthesis pathways will be discussed. Significant research efforts have been made in isolating and evaluating the biological effects of phytochemicals from the roots, stems, stem barks, leaves, and whole plant extracts of the *Suregada* species. Although different reports have been published about the medicinal uses, chemical constituents, and biological activities of *Suregada* species, currently, there is no review on the phytochemicals, pharmacological, and ethnomedicinal uses of the genus *Suregada*. Thus, our objective is to provide comprehensive insights into phytochemicals, pharmacological, and ethnomedicinal uses from *Suregada* species. Furthermore, the authors previously isolated compounds, *ent*-abietane diterpenoids, an unknown compound trivially named Mangiolide (**34**), and Jolkinolide B (**35**) from the *Suregada* species. Through a review of the literature, we aim to identify gaps in current knowledge related to *Suregada* plant species and their compounds. These identified gaps can guide future research efforts, ensuring that the scientific community focuses on areas where further investigation is most needed. The information will serve as a guideline to the researchers and assist them in focusing their research on validating the claims made on the traditional folklore uses of the *Suregada* species and/or investigations to find new phytochemicals.

## 2. Literature Search Strategy

Information about the ethnomedicinal, pharmacological activities, structure, and occurrence, of action phytochemicals from *Suregada* species was obtained from publications extracted from Science Direct, PubMed, SciFinder, Springer Link, Wiley, Scopus, Google Scholar, and Web of Science databases. The databases were searched for research articles about *Suregada* species. The syntax TITLE-ABSTRACT-KEY was used in combination with keywords like ‘*Suregada*’ OR ‘genus’ OR ‘ethnomedicinal uses’ OR ‘constituents of *Suregada*’ OR ‘biological activities of *Suregada* phytochemicals’ OR ‘antibacterial’ OR ‘cytotoxic activity’ OR ‘antiviral’, OR ‘anti-inflammatory’. The search terms were run separately or as a combination of terms depending on the database used for the period between 2000 and 2022. The search resulted in over 100 reports mostly in the English language, which we retrieved at our institution. The reports obtained information on early documents on the taxonomy and research work articles on *Suregada* since historical times as well as current reports. The obtained information from about 50 articles was carefully read, to obtain the publications meeting the aim of this work with a few older publications to reveal some necessary points. Only published work on *Suregada* was selected for this review. Due to the lack of human clinical trials, studies based on both in vitro and in vivo conditions were contained within the review, but, only those studies that used isolated substances.

## 3. Medicinal Uses of *Suregada* Species

The medicinal uses of nine of the 32 *Suregada* plants are presented in Table 1 and described below.

### 3.1. Suregada adenophora Baill.

**Figure 1:**
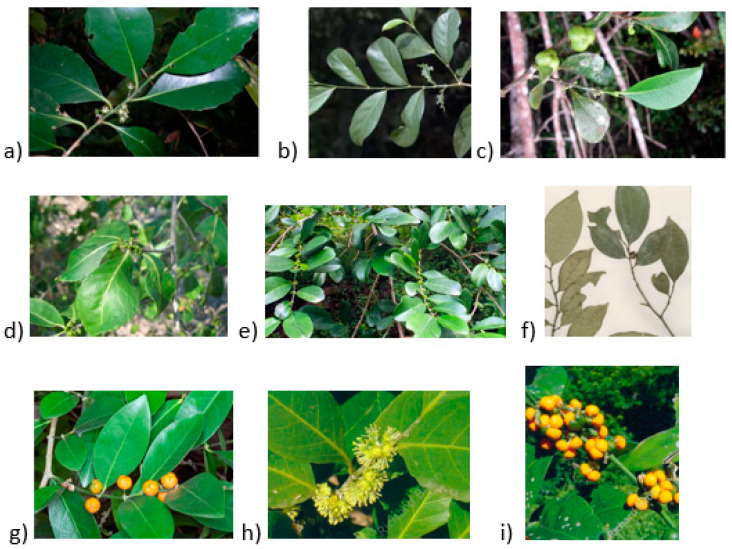
The plant species of *Suregada* (**a**) *S. adenophora*, (**b**) *S. angustifolia*, (**c**) *S. boiviniana*, (**d**) *S. decidua*, (**e**) *S. lanceolata*, (**f**) *S. lithoxyla*, (**g**) *S. multiflora*, (**h**) *S. procera*, (**i**) *S. zanzibariensis* [1,30,31,32,33].

*Suregada adenophora* (Figure 1) occurs throughout Madagascar and is used as a purgative [8].

### 3.2. Suregada angustifolia (Müll.Arg.) Airy Shaw

*Suregada angustifolia* (Figure 1) is ground and mixed with water to prepare the paste, then applied to the body for the treatment of skin infections. When the *S. angustifolia* stem bark is boiled with water and salt, it can be utilized as a mouthwash for treating toothache. Indian people in Kanis use *S. angustifolia* to treat skin infections and toothache [9].

### 3.3. Suregada boiviniana Baill. (Suregada boiviniana var. boiviniana)

*Suregada boiviniana* (Figure 1) aids in evacuating the placenta and treating dysentery, headache, cold, epilepsy, and malaria [10,11].

### 3.4. Suregada decidua Radcl.-Sm.

The fresh sap of *S. decidua* (Figure 1) from Western Madagascar is used to promote wound healing [8].

### 3.5. Suregada lanceolata (Willd.) Kuntze

The shrub of the plant is used as an astringent [12]. *S. lanceolata* (Figure 1) is used for treating skin diseases, worms, blood vomiting, piles, toothache, and weakness [13].

### 3.6. Suregada lithoxyla (Pax & K.Hoffm.) Croizat

The wood of *S. lithoxyla* (Figure 1) is hard and used to make tool handles, spoons, firewood, and poles. The tree is suitable for ornamental purposes and is used for shade [14].

### 3.7. Suregada multiflora (A. Juss.) Baill.

Jahan et al. and Choudhary et al. stated that *S. multiflora* (Figure 1) is utilized to treat gum and hepatic ailments in traditional medicines [15,16]. According to the report of Tewtrakul et al., *S. multiflorum* is mixed with other herbs and used as an anticancer recipe, while in Thailand, it is used as a traditional medicine recipe (Table 1) [17]. Tewtrakul et al., further stated that the wood of *S. multiflorum* was used to treat pyrexia, eczema, and venereal diseases; the roots of this plant are utilized to treat skin infection and lymphatic disorders [17]. In Thailand, *S. multiflora* is utilized to treat skin diseases including rashes, itching, and inflammation [18]. In some regions, the granule products of this species can be prepared, which acts as a powerful organic herbicide [19]. In India, the seeds of *S. multiflora* are utilized in the treatment of liver diseases and as a gum tonic [20]. *Suregada multiflora* is harvested as timber to be used as firewood, rafters, and tool handles and cultivated as an ornamental [21,22,23].

### 3.8. Suregada procera (Prain) Croizat

This species is suitable for musical instruments, joinery, flooring, furniture, mine props, vehicle bodies, turnery precision equipment, interior trim, novelties, sporting goods, toys, agricultural implements, and draining boards [14]. The wood of *S. procera* (Figure 1) is hard and is used for firewood, handles, and poles in construction. The tree can be used for ornaments and shade [14]. The *S. procera* stem is used to treat hemorrhoids and gonorrhea. The stem of *S. procera* is burnt and fired in the affected area [24].

### 3.9. Suregada zanzibariensis Baill.

Tanzanian people used *S. zanzibariensis* (Figure 1) stem bark and root extract for treating ankylostomiasis. Its root extract is used to treat gonorrhea, stomachache, pneumonia, hernia, chest pains, and chicken pox, and as a purgative. The roots are drunk as an extract or chewed to treat snakebites, and Kenyan people use the roots to treat edema. Crushed leaves are ingested in porridge to expel worms and to treat dysentery. The powdered leaves are consumed in porridge or tea to treat poliomyelitis [25]. In Dares Salam, *S. zanzibariensis* leaves are boiled in water and applied topically or douched two times a day to treat vaginal candidiasis [26]. Tanzanian people mix *S. zanzibariensis* leaves with the *Zanthoxylum chalybeum*, and *Acalypha fruticosa* milled and scrubbed on the skin to treat skin infection [27]. Giriama and Duruma people use a root decoction to treat body swelling. Digo people use the root decoction for body pains, for pains during menstruation, and to avoid premature birth [28,29]. The wood of *S. zanzibariensis* is hard and used for tool handles, building poles, spoons, withies, and firewood. The tree is used for shade, soil conservation near the sea, and amenity. The roots are boiled, and the juice is drank twice a day as a purgative [14].

**Table 1:** Some reported ethnomedicinal uses of *Suregada* species.

Plant	Plant Part	Uses	References
*S. adenophora*	Unspecified	Purgative	[8]
*S. angustifolia*	Stem bark	Skin infections, toothache	[9]
*S. boiviniana*	Unspecified	Purgative	[10]
	Leaves	Headaches and cold, dysentery, malaria, placenta apposition and epilepsy	[10,11]
*S. decidua*	Fresh sap	Wounds	[8]
*S. lanceolata*/*S. angustifolia*	Shrub	Astringent	[12]
	Whole plant	Skin diseases, worms, weakness, blood vomiting, piles, toothache	[13]
*S. lithoxylia*	Wood	Poles, fuel, spoons, tool handles	[14]
*S. multiflora*	Wood	Eczema, venereal diseases, and pyrexia	[17]
	Roots	Lymphatic disorders, skin diseases	
	Bark	Lymphatic disorder, hepatitis, skin diseases, fungal infection, venereal diseases, and leprosy	
	Whole Plant	Pneumonia, fever, poisonous effects, stomach disorder, squint eye, and gum disease	
*S. procera*	Wood	Poles, handles, and firewood	[14]
	Tree	Ornaments and shade	
	Stem	Hemorrhoids, gonorrhea	[24]
*S. zanzibariensis*	Leaves	Asthma, malaria, skin diseases, dysentery, vaginal candidiasis and abdominal pains	[21,26,27,29]
	Roots and stem bark	Ankylostomiasis	[25,29]
	Roots	Gonorrhoea, chest pain, stomach ache, hernia, chicken pox, schistosomiasis, body swelling, pneumonia and purgative	[14,25]
	Wood	Building poles, tool handles, withies, fuel and spoons	[14]

## 4. Phytochemical Composition of the *Suregada* Genus

**Figure 2:**
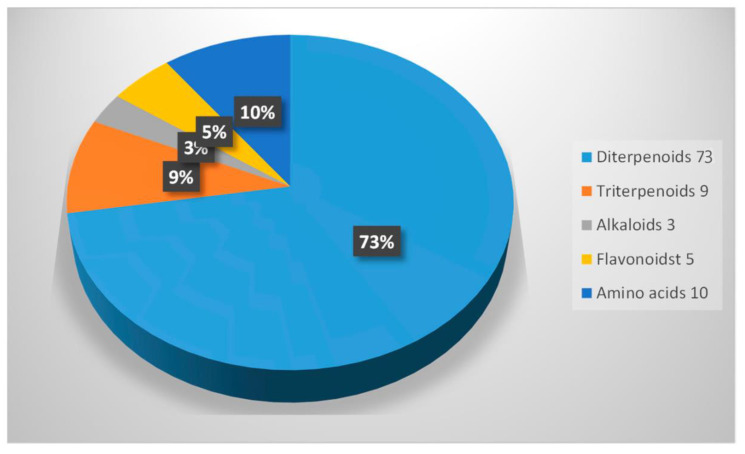
Representation of different metabolites in genus *Suregada*.

Although *Suregada* species are more valued plant species for their medicinal properties, only a few species have been studied for their ethnomedicinal and phytochemistry. There are wide discrepancies among the published results on the species’ primary metabolite content with diterpenoids frequently isolated. The literature reports 114 phytochemicals from the genus *Suregada* which were classified into diterpenoids (**1**–**71**), triterpenoids (**72**–**81**), alkaloids (**82**–**84**), flavonoids (**85**–**90**), lignans (**92**–**94**), amino acids (**95**–**104**) and iminosugars (**105**–**114**) [1,15,16,34,35] as shown in Figure 2. Diterpenoids were the major constituents reported from the genus. Most of the isolated metabolites were not evaluated for their pharmacological properties which limits their transition to clinical trials. The reports show that the phytochemical investigation of *Suregada* species has not been explored fully. Hence, future studies should aim at exploring the phytopharmacological properties of *Suregda* species.

### 4.1. Diterpenoids

Diterpenoids are a class of natural products known for their structural diversity. Diterpenoids isolated from *Suregada* genera belong to *ent*-abietane, diterpene lactone, *ent*-kuarane, and *ent*-pimaranes classes [36,37]. Ditepenoids consist of four isoprene units which form a 20-carbon backbone. The core structures for diterpenoids are classified into macrocyclic, bicyclic, linear, tricyclic, tetracyclic, and pentacyclic types [38]. Naturally, occurring diterpenoids are often found in different polyoxygenated forms with hydroxyl groups, formyl and carbonyl groups, or lactones. There are different types of diterpenoids such as *ent*-abiatane (**1**–**59**), *ent*-kaurane (**60**–**71**), etc. *Ent*-abietane diterpenoids were reported to exhibit a wide spectrum of biological activities such as cytotoxic, anti-microbial, anti-cancer, and anti-inflammatory [39]. *Ent*-Abietanes with a lactone ring are classified as one of the main bioactive compounds. For example, jolkinolide B (**35**) is well-known for its anti-tumor activity [39]. *Ent*-abietanes diterpenoids often contain an α, β-unsaturated γ-lactone ring. Some carbons of abietane diterpenoids, form a double bond or are substituted with hydroxyl or keto groups [40]. The varying skeletal structures of diterpenes result from geranylgeranyl pyrophosphate (GGPP) and are classified according to their biosynthetic pathways and cyclization patterns [41]. According to Dewick diterpenes start from geranylgeranyl diphosphate (GGPP), formed by the addition of an isopentenyl diphosphate (IPP) molecule to Farnesyl diphosphate (FPP) [41]. Cyclization of GGPP mediated by carbocation formation, allows many structural alternatives of diterpenoids to be formed [41]. Dewick further mentioned that during cyclization, the loss of diphosphate occurs which generates the first carbocation, and several natural diterpenes have been formed by varying mechanisms through cyclization [41].

The formation of carbocation formation begins by protonation of the double bond at the head of the chain, which leads to the initial cyclization sequence. Furthermore, the loss of the diphosphate later on gives rise to a carbocation and causes further cyclization. Protonation of GGPP can start a joint cyclization sequence, the loss of a proton from a methyl terminates the cyclization sequence, producing (−)-copalyl PP [41]. Folding of the substrate on the enzyme surface controls the stereochemistry of (−)-copalyl PP. Nevertheless, alternate folding results in (+)-copalyl PP (labdadienyl PP), the enantiomer of (−)-copalyl PP, which has an opposite configuration at the newly produced chiral centers. From (−)-copalyl PP, several cyclizations and a rearrangement, all catalyzed by a single enzyme kaurene synthase, produce *ent*-kaurene [42]. Formation of *ent*-kaurene involves the loss of the diphosphate leaving group which enables the carbocation-mediated product of the third ring system, which then forms the fourth ring [41].

**Scheme 1:**
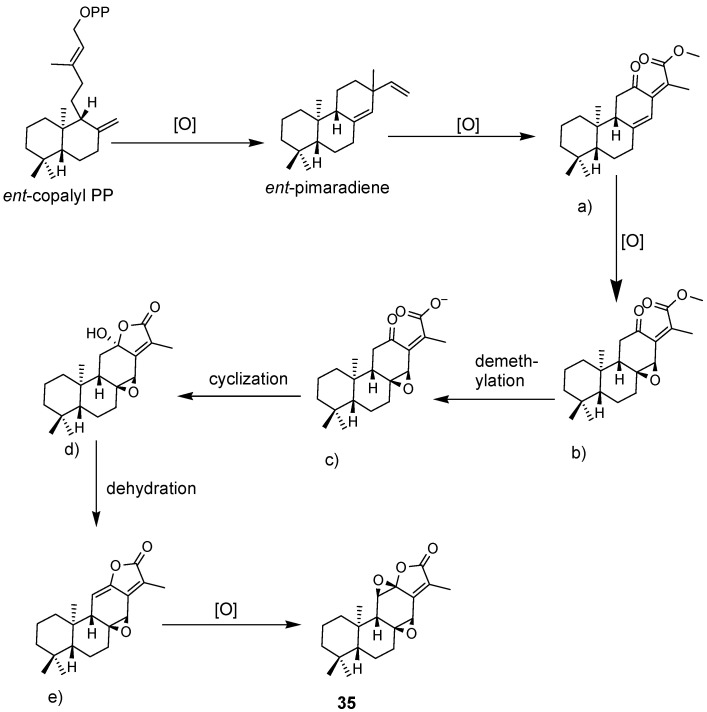
Suggested biosynthesis of some *ent*-abietane lactones including Jolkinolide B (**35**). Oxidation of *ent*-neoabietadiene led to the formation of intermediate (a). Oxidation of (a) could lead to the production of intermediate (b). Intermediate (c) was produced from a possible demethylation of (b). Intermediate see may undergo cyclization to yield (d). Dehydration of intermediate (d) produces intermediate (e).

Earlier the biosynthesis of *ent*-kaurane was described, the latter is the suggested biosynthesis of *ent*-abietane diterpenoid. The suggested biosynthesis of *ent*-abietane lactones is shown in Scheme 1 [42]. Some of the *ent*-abietane lactones including Jolikinolide (**35**) may be biosynthesized from the *ent*-neoabietadiene. The oxidation of *ent*-neoabietadiene could lead to an intermediate labeled (a). Then, the intermediate (a) might be oxidized to obtain intermediate (b) and (c) [42]. The highly oxidized *ent*-abietane diterpenoid skeleton (c) may undergo a series of intramolecular cyclization, oxidation, and dehydration reactions to produce the *ent*-abietane diterpenoids bearing an additional five-membered lactone ring, including **35** [42].

Studies conducted on *Suregada* indicated the presence of several types of diterpenoids. In particular, compounds **1**–**59**, the *ent*-abietane diterpenoids containing an additional five-membered lactone ring, revealed important pharmacological anti-tumor and anti-inflammatory activities to name a few. The specific functional groups besides the lactone ring in these ent-abietane diterpenoids can differ depending on the compound’s biosynthetic origin and its specific modifications [43]. These functional groups such as hydroxyl, epoxide, acetates, ketones, and alkene collectively contribute to the compound’s chemical and biological properties, making *ent*-abietane diterpenoids a diverse group of natural products with various potential applications in pharmacology, medicine, and other fields of research. The biological activities of *ent*-abietane diterpenoids are often related to their interactions with specific molecular targets in biological systems, and the presence of particular functional groups can be crucial for these interactions [43].

Diterpenoids are named by making use of the main name of their skeletons. If the backbone of the diterpenoid comprises many functional groups namely carboxylic, aldehyde groups, lactone, or olefinic carbons, their position will be named according to the numbering of the skeleton, and the name will be followed by the suffix -oic, -al, -olide, or -en, respectively [43]. The presence of hydroxyl and epoxide groups is named before the parent name. Greek letters α or β are utilized when a hydroxyl, epoxide, acetoxyl, or coumaroyl group in the compound is, respectively, upon or behind the plan of the skeleton [Sandjo, Rubinger] such as compounds **33**, **36**–**41**, etc. The location of epoxide, hydroxyl groups, and other substituents in the diterpene cores are given between the terms ent, syn, or neo and the name of the skeleton. The stereochemistry at C-9 and C-10 in the decalin part (ring A and B) of most diterpenoids can be cis or trans, depending on their orientation, and the naming syn and ent are utilized. When C-20 and C-11 are behind the compound plan, the prefix ent will follow the parent name [43].

**Figure 3:**
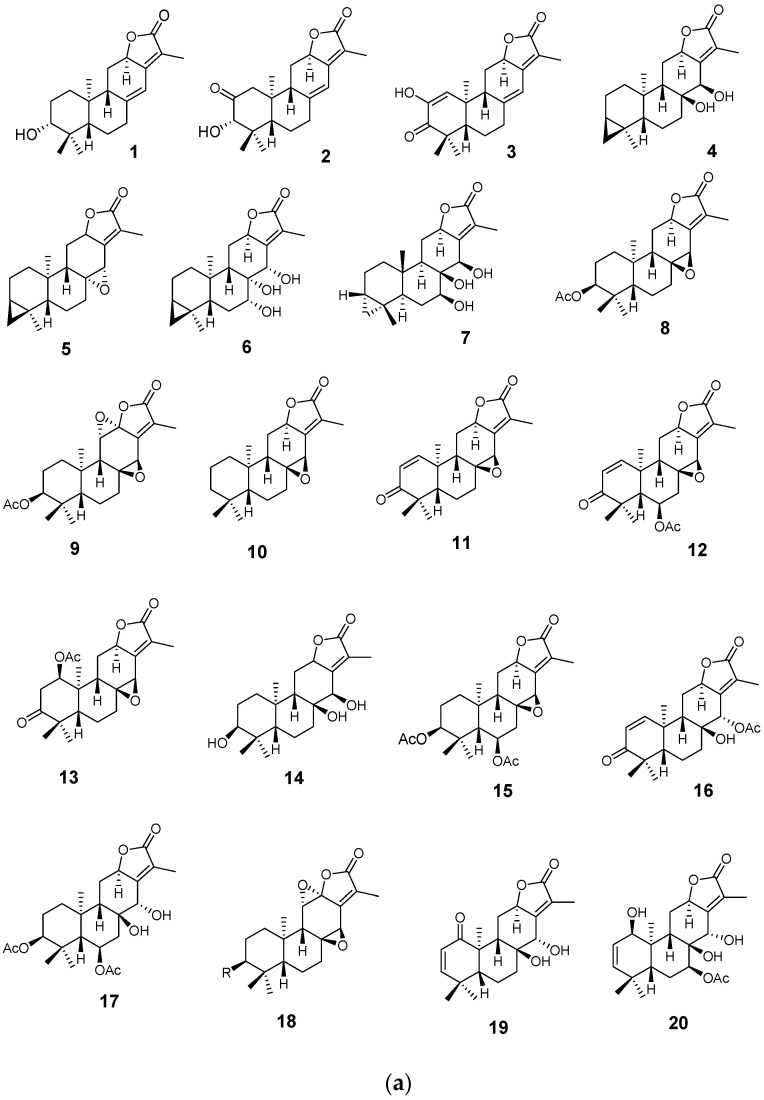

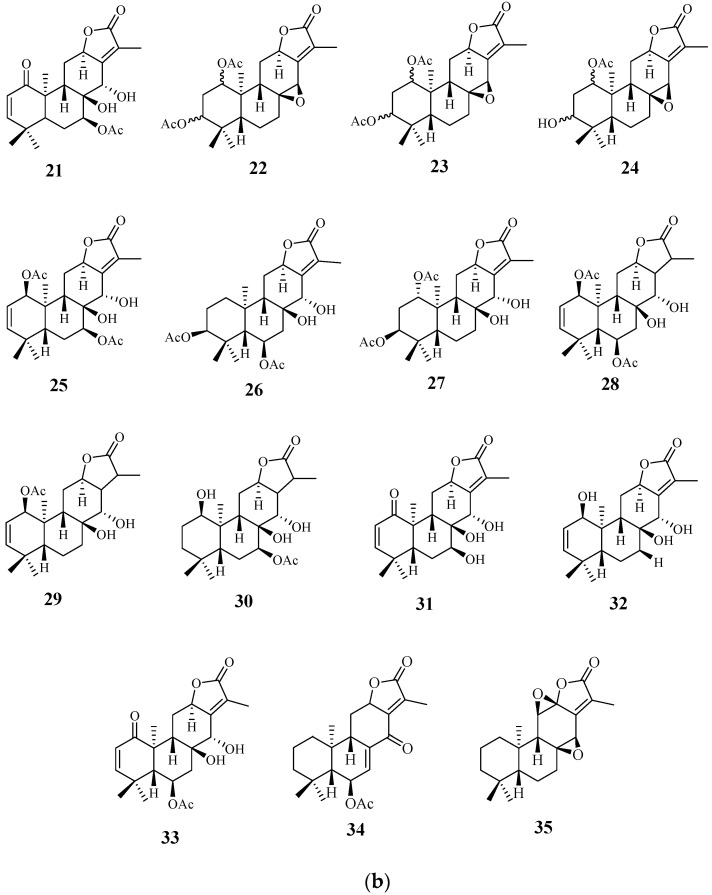

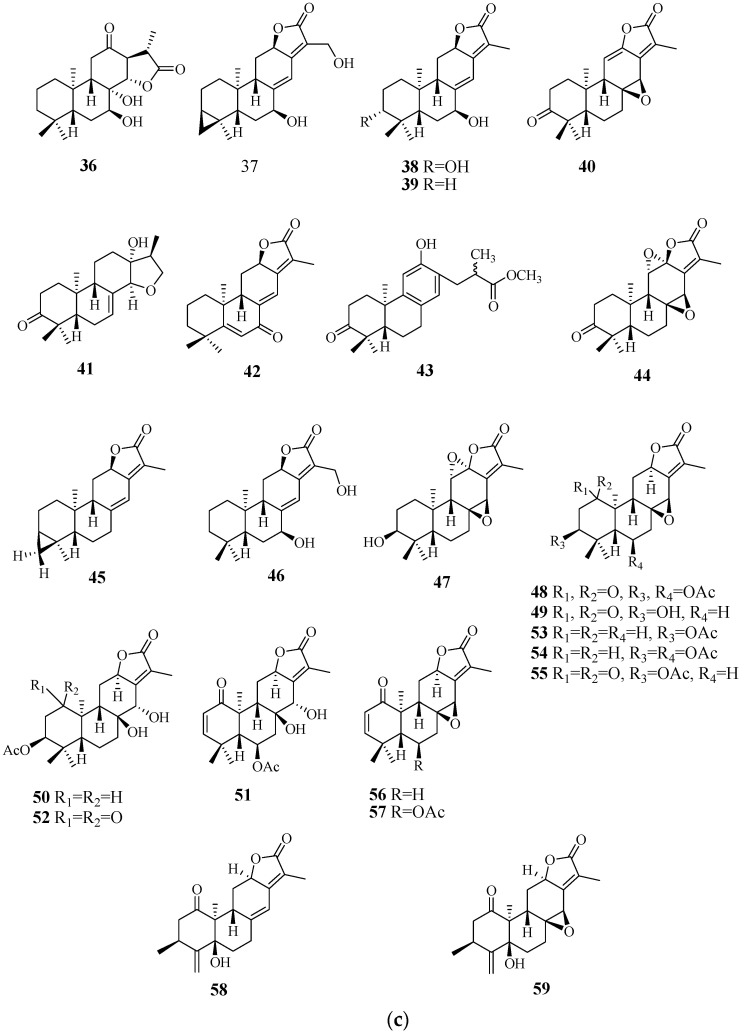
(**a**) Abiatane-type diterpenoids were isolated from *Suregada* species. (**b**) Abiatane-type diterpenoids were isolated from *Suregada* species. (**c**) Abiatane-type diterpenoids were isolated from *Suregada* species.

The number of carbons (20 signals of carbons) seen on the ^13^C-NMR spectrum could suggest that the compound is a diterpenoid. Moreover, some of the abietane, kaurane, and labdane scaffolds that do not possess any functional group on the decalin moiety such as compounds **10** and **35** exhibit carbon signals of C-1 (CH2), C-2 (CH2), C-3 (CH2), C-5 (CH), and C-6 (CH2) around δ 40.1, 18.2, 42.0, 56.0, and 20.0, respectively [44] (Figure 3c). Methyl groups C-18, 19, and 20 are seen around δ 33.1, 21.5, and 18.0, respectively. Both C-18 and 19 have Heteronuclear Multiple-Bond Correlation spectroscopy (HMBC) interactions and they correlate with C-3 (δ 42.0), C-4 (δ 33.1), and C-5 (δ 56.0) [43,44]. *Ent*-abietanes diterpenoids usually contain an α, β-unsaturated γ-lactone ring connected at C-12 and C-13. Some carbons of abietane diterpenoids, like C-8, C-14, C-11, and C-12, form a double bond or can be substituted with hydroxyl or keto groups [40]. According to the reports obtained from the search, the structure–activity relationship of compounds isolated from *Suregada* species was not evaluated. The structure–activity relationship of some reported diterpenes revealed that acetylation and esterification of hydroxyl groups, particularly at C-3 and C-8, have a positive effect on activities [40].

The ^1^H NMR spectrum of compound **4** (Figure 1) showed the presence of a trisubstituted cyclopropane ring. Two upfield resonances of the C-18 cyclopropane methylene protons H-18 (exo), δH −0.01, and H-18 (endo), δH 0.40 at δC 21.8, the H-3 signal at δH 0.54 at δC 19.1, and one quaternary carbon resonance (C-4, δC 16.1) confirmed the presence of a trisubstituted cyclopropane ring in ring A. Jahan further confirmed that these values aligned with the values reported for the metasequoic acids that were reported as a novel skeleton from the investigation of phytochemicals from Merasaquoia glyptostroboide [45] containing a trisubstituted cyclopropyl substituent in ring A of a labdane skeleton. Compounds **5**, **6**, and **7** had similar NMR signals that indicated the presence of a cyclopropane group. All these compounds were deduced to have a rearranged abietane skeleton [34]. The cyclopropyl ring on the *ent*-abietane or *ent*-abietane lactones is rare. Several modified pimarane diterpenoids and tiglianes contain the cyclopropane system [45].

**Scheme 2:**
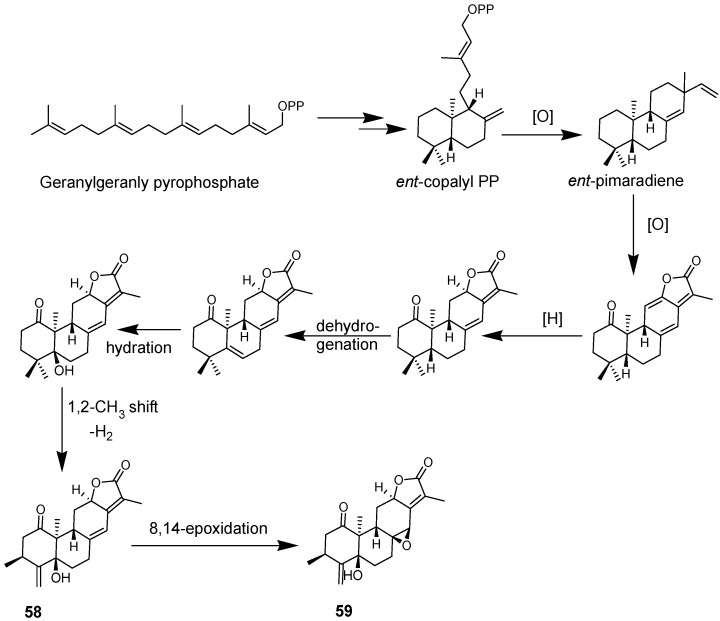
Suggested biosynthesis for compounds **58** and **59**.

Kalenga reported that modified *ent*-abietane diterpenoids with a terminal olefinic bond at C-4, such as in compounds 58 and 59, are rare. The terminal double bond at C-4 is proposed to arise through an enzymatic 1,2-methyl shift, either of CH_3_-18 or CH_3_-19, from C-4 to C-3, followed by dehydrogenation [46]. The suggested biosynthesis for compounds **58** and **59** is shown in Scheme 2 [46]. The oxidation of *ent*-copalyl PP leads to the production of *ent*-pimaradiene. Then, the *ent*-pimaradiene could be oxidized to obtain an intermediate containing a lactone ring which is an *ent*-abietane diterpenoid skeleton. The *ent*-abietane diterpenoid skeleton may undergo a series of transformations namely, hydrogenation, and dehydrogenation, and then followed by enzymatic 1.2 methyl shift to produce compound **58** or epoxidation for compound **59** [46].

The chemical structures of the diterpenoids isolated from *Suregada* species are discussed below.

### 4.2. Abiatane-Type Diterpenoids

Abietanes are tricyclic diterpenoids found in nature that have been isolated from a variety of terrestrial plant sources. Araucariaceae, Cupressaceae, Phyllocladaceae, Pinaceae, and Podocarpaceae families, as well as some species from Asteraceae, Celstraceae, Hydrocharitaceae, and Lamiaceae families, and even some fungi species, are known to contain abietane diterpenoids. Abietane diterpenoids have a wide range of biological activities. Apart from the antimicrobial, antiviral, antimalarial, antiulcer, anti-leishmaniasis, and antioxidant activities reported by the scientists, they also reveal antitumor-promoting activity and antivirus properties by inhibiting the reproduction of viruses such as herpes simplex virus type 1 (HSV-1), cytomegalovirus (CMV), varicella zoster virus (VZV), and Epstein-Barr virus [17,36,47]. Aromatic abietanes are the most abundant abietane group. Aromatic abietanes are primarily represented by dehydroabietic acid and ferruginol. Aromatic abietanes, like most diterpenoids, are mostly known as chemical defense agents. Antimicrobial, antileishmanial, antiplasmodial, antifungal, antitumor, cytotoxicity, antiviral, antiulcer, cardiovascular, antioxidant as well and antiinflammatory activities are the biological activities of this group reported up to now. Various diterpenoids have been assigned structures that could be derived by adjusting or cleaving the abietane skeleton, known as rearranged abietane. Triptergulides A and B are novel rearranged abietane diterpenes isolated from Tripterygium wilfordii (Celastraceae), a medicinal plant used in Traditional Chinese Medicine to treat a variety of diseases including systemic lupus erythematosus, psoriasis, ankylosing spondylitis, and idiopathic IgA nephropathy. Over 59 (**1–59**) *ent*-abietane-type diterpenoids were isolated from *Suregada* species which remains the abundant diterpenoids from the genus.

*Ent*-abietane and *ent*-kuarane diterpenoid lactones were isolated from *S. multiflora*, *S. glomerulata*, *S. aequorea* and *S. zanzibariensis*. The dichloromethane extract of *S. multiflora* bark afforded three tetracyclic diterpene lactones; helioscopinolide A (**1**), helioscopinolide C **(2**) and helioscopinolide I (**3**) (Figure 1a) [17,36]. Suregadolides A and B (**4** and **5**) were isolated from the dichloromethane extract of *S. multiflora* bark (Figure 1a). Dichloromethane-methane extract of *S. multiflora* bark afforded suregadolides C (**6**) and D (**7**) (Figure 1a) these compounds were found to possess a novel skeleton, containing a cyclopropane ring bridging at C-3 and C-4 of the abietane skeleton [16,34]. Gelomulides A-F (**8**–**13**) were isolated from the *S. multiflorum* leaves (Figure 1a) [48]. *S. multiflora* leaves were reinvestigated and led to the isolation of Suremulide A (**14**) and gelomulides G-J (**15**–**18**) (Figure 3a) [15,16,34,49].

Lee et al. reported the isolation of *ent*-abietane diterpenes, namely, gelumolide K-X (**19**–**32**) (Figure 3a,b) and 6β-acetoxy-1-one-8β,14-α-dihydroxy-*ent*-abieta2(3),13(15)-diene,12-olide (**33**) [35] from the dichloromethane extract of *S. aequorea*.

Mangisa et al., isolated 6-acetoxy-14-keto-*ent*-abieta-7(8),13(15)-diene-16,12-olide) trivially named mangiolide (**34**) and 8,13-diepoxy-13,15-*ent*-abietene-16,12-olide, Jolkinolide B (**35**) from the dichloromethane-methanol extract of *S. zanzibariensis*, (Figure 3b) [50].

*Ent*-abiatene diterpenoids 7β,8αβ-dihydroxy-12-oxo-*ent*-abietan-16,14-olide (**36**), 3,4,18β-cyclopropa-7β,17-dihydroxy-*ent*-abieta-8(14),13(15)-dien-16,12-olide (**37**), 3α,7β-dihydroxy-*ent*-abieta-8(14),13(15)-dien-16,12-olide (**38**), 7β-hydroxy-ent-abieta-8(14),13(15)-dien-16,12-olide (**39**), 3-oxo-8β,14β-epoxy-*ent*-abieta-11,13(15)-dien-16,12-olide (**40**), 13α-Hydroxy-14α,16-epoxy-*ent*-abieta-7-en-3-one (**41**), 7-Oxo-*ent*-abieta-5,8(14),13(15)-trien-16,12-olide (**42**), Methyl 12-hydroxy-3-oxo-*ent*-abieta-8,11,13-trien-17-oate (43), 3-oxo-jolkinolide B (**44**), 3,4,18b-cyclopropa-*ent*-abieta-8(14),13(15)-dien-16,12-olide (**45**) and 7β,17-dihydroxy-*ent*-abieta-8(14),13(15)-dien-16,12-olide (46) were isolated from the ethanol extract of *S. glomerulata* roots (Blume) Baill. [51,52,53].

Dichloromethane-methanol extract of *S. multiflora* leaves afforded 3β-Hydroxy-8,14β:11,12α-diepoxy-13(15)-abietane-16,12-olide (**47**) [53]. Investigation of methanol-dichloromethane (1:1) extract of *S. multiflora* leaves afforded ten diterpene lactones namely, 3β,6β-diacetoxy-1-one-8β,14β-epoxy-13,15-abiatene-16,12-olide (**48**), 3β-hydroxy-1-one-8β,14β-epoxy-13,15-abiatene-16,12-olide (**49**), 3β-acetoxy-8β,14α-dihydroxy-13,15-abiatene-16,12-olide (**50**), 6β-acetoxy-2-ene-8β,14α-dihydroxy-1-one-13,15-abiatene-16,12-olide (**51**), 3β-acetoxy-8β,14α-dihydroxy-1-one-13,15-abiatene-16,12-olide (**52**), 3β-acetoxy-8β,14β-epoxy-13,15-abiatene-16,12-olide (**53**), 3β,6b-diacetoxy-8β,14β-epoxy-13,15-abiatene-16,12-olide (**54**), 3β-acetoxy-1-one-8β,14β-epoxy-13,15-abiatene-16,12-olide **(55**), 2-ene-1-one-8β,14β-epoxy-13,15-abiatene-16,12-olide (**56**), and 6β-acetoxy-2-ene-1-one-8β,14β-epoxy-13,15-abiatene-16,12-olide (**57**) [15]. *S. zanzibariensis* leaf extract yielded zanzibariolides A (**58**) and B (**59**) [46].

### 4.3. Kaurene-Type Diterpenoids

**Figure 4:**
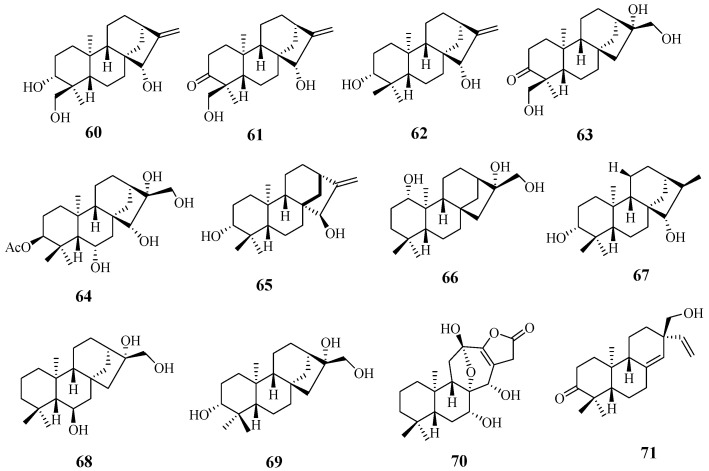
Structures of kaurene and *ent*-pimarane-type diterpenoids from *Suregada* species.

Cheenpracha et al., Jahan et al., and Tewtrakul et al. investigated the active dichloromethane extract of the *S. multiflora* stem bark which yielded several diterpenoids *ent*-kaurene diterpenoids, *ent*-16-kauren-3β,15β,18-triol (**60**), and *ent*-3-oxo-16-kauren-15β,18-diol (**61**) together with *ent*-16-kaurene-3β,15β-diol (**62**), and abbeokutone (**63**) (Figure 4) [16,17,36]. According to Gondal, Jahan et al., and Tewtrakul et al., Suremulol A (**64**) and Suremulol B (**65**) were also isolated from *S. multiflora* (Figure 2) [16,17,51]. He et al., and Yan et al., stated that the ethanol extract of *S. glomerulata* roots yielded 1α,16α,17-Trihydroxy-*ent*-kaurane (**66**), euphoranginol B (**67**), corymbol (**68**), and *ent*-kaurane-3β,16β,17-triol (**69**) (Figure 4) [52,53].

### 4.4. Ent-Pimarane Diterpenoids

**Figure 5:**
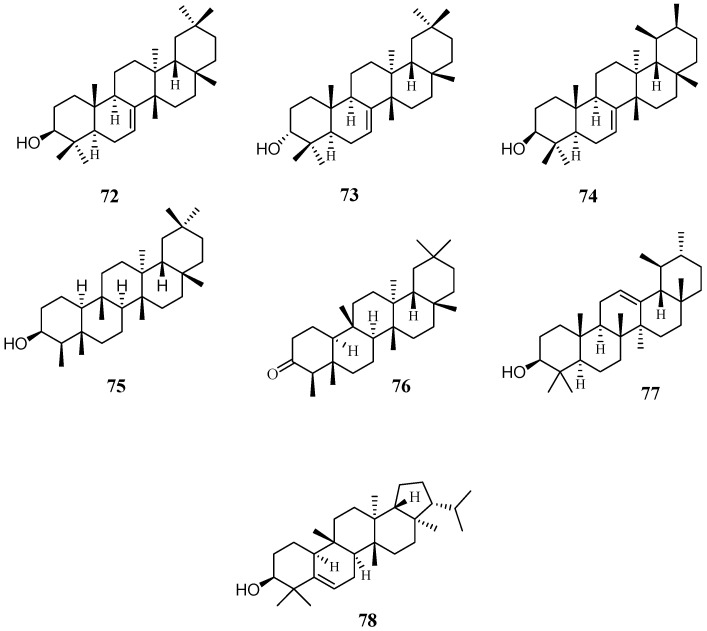
Structures of triterpenoids isolated from *S. multiflora*.

Jahan et al. investigated *S. multiflora* which yielded bannaringaolide A (**70**) (Figure 5) [16]. A diterpene lactone of an *ent*-pimarane skeleton, 17-hydroxy-*ent*-pimara-8(14),15-dien-3-one (**71**) was isolated from the ethanol extract of *S. glomerulata* roots [52].

### 4.5. Triterpenoids

**Figure 6:**
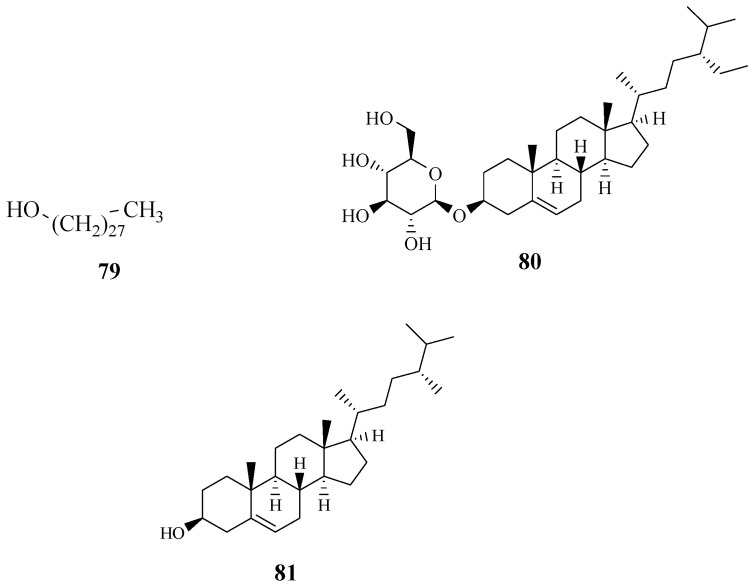
Structures of fatty alcohol, steroidal glycoside, and phytosterols isolated from *S. angustifolia*.

Sengupta and Khastgir reported the isolation of three triterpenoids, namely: 7-multifloren-3β-ol (**72**), 7-multifloren-3α-ol (**73**), and baurenol (**74**) (Figure 6) from the bark extract of *S. multiflora* [54]. Baurenol (**74**) was also isolated from *S. angustifolia*. Venkatesan et al. reported six compounds, epi-friedelinol (**75**), friedelin (**76**), and α-amyrin (**77**) from *S. angustifolia* stem bark [9]. α-amyrin (**77**) and simiarenol (**78**) were also isolated from *S. zanzibariensis* leaves extract [46].

### 4.6. Fatty Alcohol, Steroidal Glycoside and Phytosterol

n-Octacosanol (**79**), β-sitosterol-3-β-D-glucopyranoside (**80**), and β-sitosterol (**81**) were isolated from *S. angustifolia* stem bark [9].

### 4.7. Alkaloids

**Figure 7:**
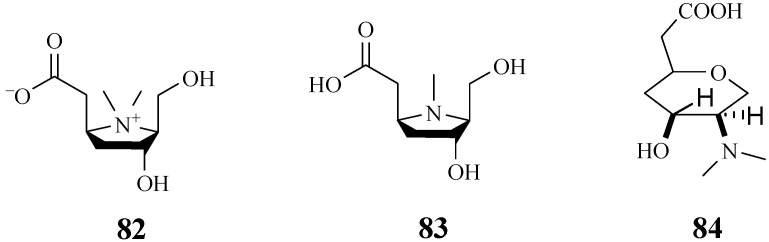
Structures of alkaloids isolated from *S. glomerulata*.

Yan et al. explored *S. glomerulata* leaves (Blume) Baill., which afforded three pyrrolidine alkaloids, namely 5β-carboxymethyl-3α-hydroxy-2β-hydroxymethyl-1,1-dimethylpryrrolidine (**82**), 5β-carboxymethyl-3α-hydroxy-2β-hydroxymethyl-1-methylpryrrolidine (**83**) and 2α-carboxymethyl-5α-dimethylamino-4α-hydroxy-tetrahydrpyran (**84**) (Figure 7) [52]. Polyhydroxyalkaloids such as trimethylsilyl (TMS) derivatives of α-homonojirimycin were isolated from *S. gaultheriifolia* Radcl.-Sm leaves and *S. adenophora* leaf fragment [55].

### 4.8. Flavonoids

**Figure 8:**
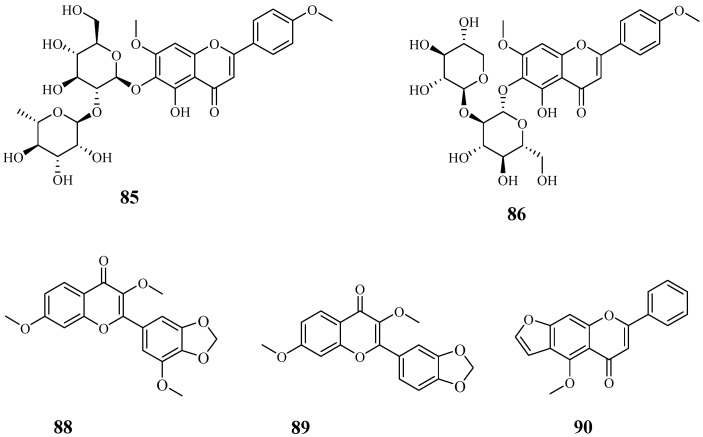
Structures of flavones isolated from *S. multiflora*.

**Figure 9:**
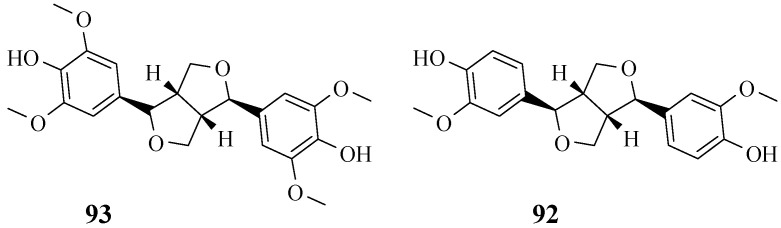
Structures of lignans isolated from *S. multiflora*.

Flavone-type flavonoids have been isolated from *Suregada* species [56]. Flavone glycosides, gelomuloside A (**85**) and B (**86**); flavone glycoside, 7,4’-*O*-l-ramnopyranosyl (12)-d-glucopyranoside (**87**) (Figure 8), resulted from the investigation of seeds of *S. multiflora*. Three other flavones were obtained from the isolation of the leaves of *S. multiflora*, namely, kanugin (**88**), dimethoxy kanugin (**89**), and pinnatin (**90**) (Figure 8) [57]. Das and Chakravarthy reported the isolation of a flavone glycoside, luteolin-7,4′-dimethyl ether 3′-glucoside (**91**) from the *S. multiflora* leaf extract (Figure 9) [57,58].

### 4.9. Lignans

Gondal and Choudhary isolated two lignans, pinoresinol (**92**) and syringaresinol (**93**), from the methanol extract of leaves of *S. multiflora* (Figure 9) [15,47].

### 4.10. Amino Acids

**Figure 10:**
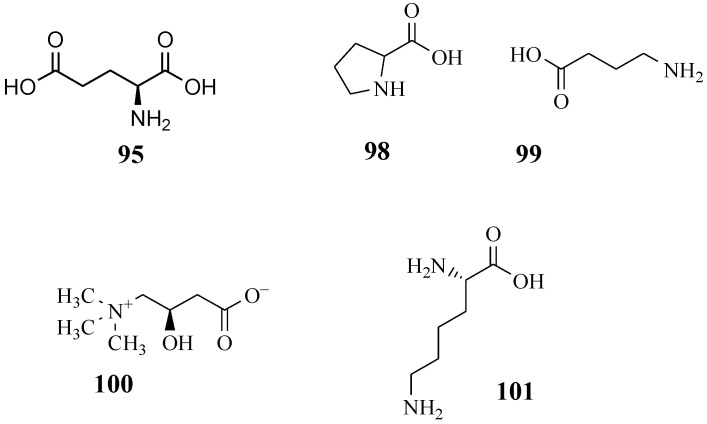
Structures of amino acids isolated from *S. glomerulata*.

The leaves of *S. glomerulata* were investigated and afforded twelve amino acids, namely, alanine (**94**), glutamic acid (**95**), leucine (**96**), isoleucine (**97**), proline (**98**), γ-aminobutyric acid (**99**), (R)-carnitine (**100**), lysine (**101** (Figure 10) [53].

### 4.11. Pyrimidines, Purine Nucleoside and Pyridines

**Figure 11:**
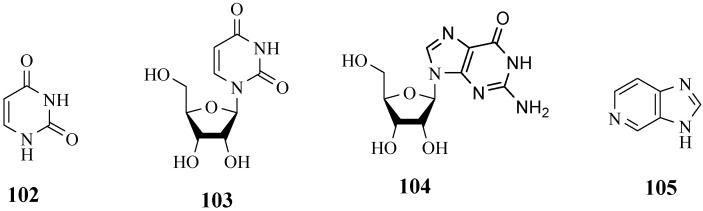
Structures of pyrimidines, purine nucleoside, and pyridines isolated from *S. glomerulata*.

Uracil (**102**), uridine (**103**), guanosine (**104**), and 3H-imidazo [4,5-c]pyridine (**105**) (Figure 11) were isolated from The leaves of *S. glomerulata* [53].

### 4.12. Pyrrolidine-Type Iminosugars

**Figure 12:**
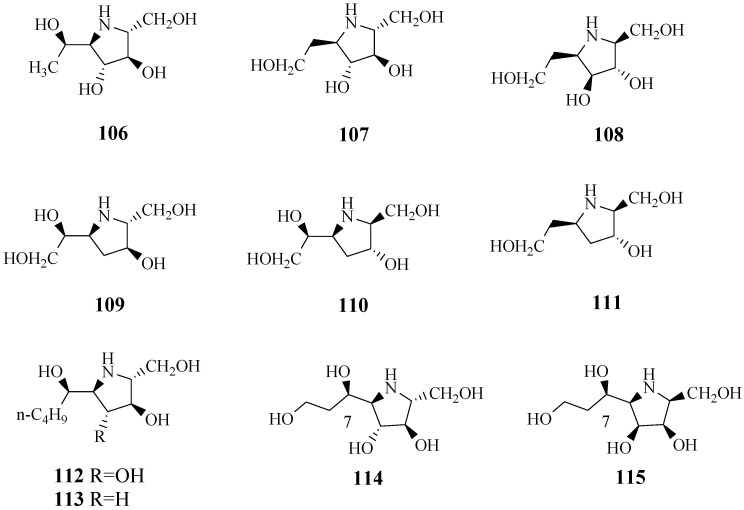
Structures of pyrrolidine-type iminosugars isolated from *S. glomerulata*.

The water extract of *Suregada glomerulata* leaves afforded ten pyrrolidine-type iminosugars, namely: 7-deoxy-homoDMDP (**106**), 6-deoxy-homoDMDP (**107**), 2,5-imino-2,5,6-trideoxy-D-gulo-heptitol (**108**), 2,5-imino-2,4,5-trideoxy-D-manno-heptitol (**109**), 2,5-imino-2,4,5-trideoxy-D-gulo-heptitol (**110**), 2,5-imino-2,4,5,6-tetradeoxy-D-gulo-heptitol (**111**), 6-C-butyl-DMDP (**112**), 6-C-butyl-4-deoxy-DMDP (**113**), 6-C-(8-hydroxyoctyl)-DMDP (**114**) and 6-C-(8-hydroxyoctyl)-2,5-dideoxy-2,5-imino-D-galactitol (**115**) (Figure 12) [59].

## 5. Pharmacological Activities of the *Suregada* Genus

The reviewed published article revealed that some of the isolated phytochemicals were tested for their pharmacological activities while some were not evaluated. *Suregada* species and some phytochemicals were screened for their antileishmanial, antidiabetic, antioxidant, cytotoxic, anti-plasmodial, antimicrobial, and anticancer activity. A summary of some *Suregada* species activities is presented in Table 2.

### 5.1. Antibacterial and Antimicrobial Activity

Venkatesan et al., reported the screening of methanol, chloroform, and hexane extracts of *S. angustifolia* stem bark at various concentrations (20, 10, and 5 mg·mL^−1^) against twelve human pathogenic bacteria [9]. The maximum zone inhibition at 20 mg·mL^−1^ in chloroform extract was observed as *Klebsiella pneumonia* (37 mm), *Staphylococcus aureus* (32 mm), A. hydrophila (40 mm), and *Escherichia coli* (40 mm). In comparison, the observations at 10 mg·mL^−1^ were slightly different observed as *E. coli* (32 mm), *K. pneumoniae* (30 mm), *S. aureus* (25 mm), and *A. hydrophila* (33 mm) [9]. Hexane extract at 20 mg·mL^−1^ revealed inhibition against *K. pneumonia* (29 mm), *A. hydrophila* (30 mm), *Proteus vulgaris* (25 mm), *V. parahaemolyticus* (25 mm), *V. vulnificus* (25 mm) and *V. cholera* (25 mm). The methanol extract at 20 mg·mL^−1^ showed zone inhibition against *S. aureus* (32 mm), *E. aerogenes* (26 mm), *E. coli* (26 mm), and *P. vulgaris* (25 mm) [9].

Aqueous, chloroform, and ethanol extracts of *S. angustifolia* bark and leaves were screened against two bacterial strains (Gram-positive and negative) and two fungal strains using the disc diffusion method. The aqueous leaf extract revealed zone inhibition of *Pseudomonas aeruginosa* (18.00 mm) and *Bacillus subtillis* (8.00 mm). The aqueous extract of *S. angustifolia* bark revealed zone inhibition of *P. aeruginosa* (20.00 mm) and *B. subtillis* (15.00 mm). The chloroform bark extract showed antibacterial properties against *P. aeruginosa* and *B. subtilis* with zone inhibition values of 18.00 mm and 15.00 mm. The ethanol leaf extract revealed activity against *P. aeruginosa*, *S. aureus*, and *B. subtilis* with zone inhibition values of 9.00 mm, 20.00 mm, and 12.00 mm, respectively [25].

The antibacterial activity of *S. multiflorum* leaves, bark, and stem extracts was investigated. The leaves hexane extracts revealed partial zone inhibition against *Mycobacterium lacticola* and *S. aureus* (10 to 11 mm). The hexane extract of *S. multiflorum* bark showed zone inhibition of 10 mm against *S. aureus*. The leaf dichloromethane extracts partially showed zone inhibition of 10 mm against *S. aureus*, 0.5 mm against *E. coli*, and 11 mm against *M. lacticola* and moderately revealed zone inhibition of 16 mm *Bacillus subtilis* [60]. The bark dichloromethane extracts showed a moderate zone inhibition of 12 to 13 mm, 15 mm, 13 to 14 mm, and 12 to 13 mm against *S. aureus*, *B. subtilis*, *M. lacticola*, and *Xanthomonas campestris*, respectively. The stem hexane extract revealed partial inhibition against *M. lacticola* with a zone inhibition value of 11 mm. The stem-dichloromethane extracts showed partial zone inhibition of 0.3 mm against *E. coli* and 3 mm against *X. campestris* and moderate inhibition against *S. aureus*, *B. subtilis*, and *M. lacticola* with zone inhibition of 13 mm, 13 mm and 18 mm, respectively [60]. The dichloromethane extract was the most active extract, followed by hexane and methanol extracts [60].

The (1:9) methanol-ethyl acetate extract of *S. multiflora* leaves and root was investigated against four gram-negative and five gram-positive bacterial strains at a dose of 200 µg.disc-1. *Suregada multiflora* root extract revealed a promising antimicrobial effect against *B. subtilis*, *Shigella boydii*, *E. coli*, *S. aureus*, *B. cereus*, *B. megatherium*, *B. anthracis*, *Shi. flexneri* and *P. aeruginosa* with the zone inhibition values of 12 ± 0.05 mm, 12 ± 0.07 mm, 13 ± 0.01 mm, 12 ± 0.07 mm, 11 ± 0.13 mm, 10 ± 0.22 mm, 11 ± 0.02 mm, 10 ± 0.01 mm, 11 ± 0.12 mm, and 9 ± 0.05 mm, respectively, with E. coli being the highest [61]. The *S. multiflora* leaf extract showed mild potency when screened against *B. subtilis*, *S. aureus*, and *Shi. flexneri*, *E. coli*, and *Pseudomonas aeruginosa* with the zone inhibition range of 5 ± 0.05 to 6 ± 0.05 mm [62]. Since significant activity was observed in all the tested bacterial strains for the root extract, minimum inhibitory concentration (MIC) values were only applied to the root extract. *E. coli* was the most sensitive, with a MIC of 0.625 mg/mL [61]. *S. multiflora* root extract further revealed a maximum relative percentage inhibition against *Shi. boydii* (34.45%), *B. subtilis* (32.12%) and *E. coli* (41.23%) at 200 µg·mL^−1^ dose. The leaf extract of *S. multiflora* showed a relative percentage range between 11.21 and 14.53%, and the applied dosage was 200 µg·mL^−1^ [61].

Helioscopinolide A (**1**) isolated from *S. multiflora* (**1**) showed an antibacterial effect against *S. aureus* 6538P (2.5 µg·spot^−1^) [62]. Epifriedelinol (**75**) isolated from *S. multiflora* was previously screened against seven Gram-negative bacterial strains, namely, *K. pneumoniae*, *P. aeruginosa*, *Salmonella typhimurium*, *E. coli*, *and Shi. Flexneri*, *Proteus vulgaris*, and *Shi. sonnei* and five Gram-positive bacterial strains, namely *B. subtilis*, *B. pumilus*, *B. cereus*, *Micrococcus luteus* and *S. aureus* [59]. Epifriedelinol (**75**) revealed zone inhibition Grange of 14.00 ± 0.58 to 25.66 ± 0.88 mm against all the Gram-positive and negative bacterial strains with the MIC range of 6.25 and 50 µg·mL^−1^ and the minimum bactericidal concentration (MBC) range of 12.5 and 100 µg·mL^−1^. Epifriedelinol (**75**) was the most sensitive against *S. aureus*, with a zone inhibition of 25.66 ± 0.58 mm. MBC of 12.5 µg·mL^−1^ and MIC of 6.25 µg·mL^−1^. Epifriedelinol (**75**) revealed lower activity against *K. pneumoniae* with a zone inhibition value of 14 ± 0.88 mm. MBC of 100 µg·mL^−1^ and MIC of 50 µg·mL^−1^ [60,63].

### 5.2. Insecticidal Activity

The (1:9) methanol: ethyl acetate extract of *S. multiflora* root revealed a 100% mortality rate for *Tribolium castaneum* at 50 mg·mL^−1^ dose in 12 h, while leaves extract revealed a mortality rate of 40% for *Tribolium castaneum* at 50 mg·mL^−1^ dose in 48 h. Strong insecticidal activity was observed on the *S. multiflora* root extract. A dose-dependent manner was carried out, where five graded doses, namely, 5, 10, 20, 30, and 40 µg·mL^−1^ were utilized, and the mortality rate was 33.33%, 53.33, 86.66, 93.33, and 100, respectively [61].

### 5.3. Antifungal Activity

Jahan et al. stated that Suregadolide A (**4**) isolated from *S. multiflora* revealed an antifungal effect of minimum concentration that produces zone inhibition of 12 mm diameter (IC_12_) of 70 and 35 µg·mL^−1^ in the RAD+ and RAD52 mutant yeast assays, respectively [34].

### 5.4. Anticancer Activity

In 2008, Lee et al. published that the dichloromethane extract of *S. aequoreum* was active against several human cancer cells with IC_50_ < 20 µg·mL^−1^ [36]. Yan et al. stated that *S. glomerulata* roots were tested against five tumor human cancer cells, namely, liver cancer (Bel 7402), ovarian (A2780), lung cancer (A549), stomach (BGC 823) and colon (HCT-8) and showed weak cytotoxic effects against all the cancer cells [54]. *S. zanzibariensis* stem bark (1:1) dichloromethane-methanol extract was screened at 100 µg·mL^−1^ dose to evaluate anticancer properties against melanoma (UAC62), breast (MCF7) and cells renal (TK10). The (1:1) dichloromethane-methanol extract was potent against TK10, UACC62 and MCF7 cancer cells with the Total Growth Inhibition (TGI) of 0.60 µg·mL^−1^, 0.54 µg·mL^−1^ and 5.27 µg·mL^−1^, respectively, and 50% Growth Inhibition (GI_50_) of 0.25 µg·mL^−1^ for UACC62, 0.26 µg·mL^−1^ for TK10 and 0.81 µg·mL^−1^ for MCF7 [51]. Helioscopinolide A (**1**) was potent against breast adenocarcinoma (MCF-7) and lung cancer (NCI-H460) with GI_50_ of 67.50 ± 3.04 µM and 72.78 ± 6.33 µM [64] and inhibited anticancer activity against human cervical carcinoma (HeLa) (IC_50_ = 0.11 µM) and breast cancer (MDA MB-231) (IC_50_ = 2.1 µM) cells [65]. Gelomulide E (**12)** isolated from *S. multiflora* was potent at a concentration of 5x10-5 M against the lung (NCI H490) cancer cell with growth inhibition of more than 85% [62]. Gelomulide E (**12**) was screened in a panel of 60 tumor cells and was reported to show activity against leukemia (CCRF-CEM), leukemia (SR), leukemia (K-562), breast (MD MB-435), and colon (HTC-15) with more than 95% growth inhibition [56].

Gelomulide K (**19**) and M (**20**) isolated from *S. aequorea* afforded moderate cytotoxicity activities against breast (MCF7 and MB-231), liver (HepG2), and lung (A549) with the IC50 range of 10.5 to 29.8 µM [35]. Gelomulide K (**19**) suppressed the growth of breast cancer cells, such as MDA-MB-231, BT474, MCF-7, MDA-MB-468 and SKBR3, with the IC_50_ range of 25.30 to 37.84 µmol·L^−1^ [49]. Mangiolide **(34**) isolated from *S. zanzibariensis* exhibited anticancer activities with TGI values of 0.02 µg·mL^−1^ (TK10), 0.03 µg·mL^−1^ (UACC62) and 0.05 µg·mL^−1^ (MCF7) and the GI_50_ values 0.07 µg·mL^−1^ (TK10); 0.06 µg·mL^−1^ (UACC62) and 0.33 µg·mL^−1^ (MCF7). Jolkinolide B (**35**) isolated from *S. zanzibariensis* showed anticancer properties with the TGI of 13.99^,^ 5.03 and 62.03 µg·mL^−1^ against TK10, UACC62 and MCF7 cancer cells, respectively, and GI_50_ values of 0.94 µg·mL^−1^ for UACC62, 3.31 µg·mL^−1^ for TK10 and 2.99 µg·mL^−1^ for MCF7 and further exhibited selectivity against melanoma (UACC62) [50].

Jolkinolide B (**35**) reduced the production of lactic acid and ATP and induced tumor cell apoptosis in mouse melanoma B16F10 cells. Jolkinolide B (**35**) showed a low level of the mRNA expression of glycolysis-related kinase genes (Ldha and Hk2) in B16F10 cells and glucose transporter genes (Glut1, Glut3, and Glut4) [66,67,68]. Jolkinolide B (**35**) showed an increased level of the mRNA expression of pro-apoptosis genes (Bax) and reduced the rate of the mRNA expression of anti-apoptosis genes (Caspase-3, Caspase-9, and Bcl-2) which confirms its anticancer effect. It improved reactive oxygen species (ROS) levels in B16F10 cells and reduced the mitochondrial membrane potential [65,66,67]. Jolkinolide B (**35**) was reported to induce the tumor apoptosis of murine melanoma B16F10 cells in a mouse xenograft model and suppress tumor growth. Jolkinolide B (**35**) induced apoptosis viability and decreased cells in a time- and dose-dependent manner in human leukemic (U937) [62]. Jolkinolide B **(35**) decreased the colony formation and cell viability of HT29, CRC, and SW620 cells [65,66,67]. Jolkinolide B (**35**) showed phosphorylation of extracellular signal-regulated kinase (ERK) [68]. According to Luo et al., Jolkinolide B (**35**) showed the linkage of MDA MB 231 cells to fibronectin [68]. Jolkinolide B (**35**) reduced the proliferation of three human cancer cells, namely, oesophageal carcinoma (Aca-109), chronic myeloid leukemia (K562), and hepatoma (HepG2), with the IC_50_ of 23.7 µg·mL-1, 12.1 µg·mL^−1^ and >50.0 µg·mL^−1^, respectively [69]. 6β-acetoxy-2-ene-1-one-8β,14β-epoxy-13,15-abiatene-16,12-olide (56) demonstrated promising anticancer effect against the lung (NCI-H460) cell with 85% growth inhibition at 5 × 10^−5^ M. Compound (56) further activity against Leukemia (SR, CCRF-CEM and K-562), Breast (MD-MB-435) and Colon (HCT-15) and with over 95% growth inhibition [15].

Bauerenol (**74**) exhibited apoptosis-inducing potential against HepG2 cancer cells and growth-inhibitory effects. Bauerenol (**74**) inhibited the proliferation of retinoblastoma cells (SO-Rb50), with the IC_50_ of 10 μM (*p* < 0.05). Furthermore, bauerenol (**74**) suppressed the invasion and movement of SO-RB50 cells by (*p* < 0.05) [70].

### 5.5. Antinociceptive Activity

Simiarenol (**81**) isolated from *S. zanzibariensis* exhibited a noticeable antinociceptive effect with the 50% infectious dose (ID_50_) of 18.87 (14.6–24.4) mmol·kg^−1^. These results were more active compared to other reference drugs, such as dipyrone and aspirin, which are known as anti-inflammatory and analgesic drugs, with an ID_50_ of 133 and 162 mmol·kg-1, respectively [71,72].

### 5.6. Antidiabetic Activity

The water extract of *S. glomerulata* leaves revealed an inhibitory effect against α-glucosidase with the IC_50_ 2.29 µg·mL^−1^ [53].

### 5.7. Antiviral Activity

*Suregada multiflora* revealed the presence of anti-human immunodeficiency virus-1 (HIV 1) protein, glycosylation-associated protein (GAP3), and displayed an inhibitory effect on the replication and infection of anti-HIV activity, and herpes simplex virus (HSV) [15,73]. *S. zanzibariensis* leaf extract revealed toxicity against herpes simplex virus 2 (HSV-2) with the IC_50_ of 11.5 µg·mL^−1^ and toxic on African green monkey kidney cells (GMK AH1) with the CC_50_ 52 µg·mL^−1^. Zanzibariolides A (**58**) and B (**59**) and simiarenol (**81**) exhibited no anti-HSV-2 effect and displayed minor toxicity against GMK AH1 cells at ≥100 µM [45].

### 5.8. Cytotoxicity

The dichloromethane-methanol crude extract of *S. multiflorum* revealed promising cytotoxicity against 60-cell tumour panels conducted at the NCI [16]. According to Kigondua et al., the ethyl acetate extract of *S. multiflorum* stem was cytotoxic against the cancer cell (MDA-MB435) [74]. Ethanol extract of the *S. multiflorum* bark revealed cytotoxicity against cervical cancer (Hela cells). The leaves aqueous and methanol extracts of *S. zanziberiensis* revealed low toxicity towards human embryonic lung fibroblast (HELF) cells with a 50% cytotoxic concentration (CC_50_) of >20 µg·mL^−1^ [74].

Luo et al. reported that Jolkinolide B (**35**) revealed weak cytotoxicity against five human cancer cells, namely, Bel 7402, A549, (BGC 823), HCT-8, and A2780 with the IC_50_ of 5.95, 6.10, 5.84, 6,88, and 5.09 µg·mL^−1^, respectively [70]. 3-oxo-jolkinolide B (**44**) showed a moderate cytotoxic effect against HCT-8, A549, BGC 823, Bel 7402, and A2780 with the IC_50_ of 6,77, 8.03, 10, 7.20, and 6.81 µg·mL^−1^, respectively [64]. The treatment of HepG2 cells with bauerenol (**74**) reduced the growth significantly with a 50% growth inhibitory concentration of 25 µg·mL^−1^ [75].

### 5.9. Anti-Inflammatory

The stem bark of *S. multiflora* showed anti-inflammatory activity against lipopolysaccharide (LPS)-induced nitric oxide (NO) and prostaglandin E(2) (PGE(2)) releases in RAW264.7 cells [17]. Helioscopinolide A (**1**) was the most active compound against NO release with an IC_50_ of 9.1 μM than helioscopinolide C (**2**) with an IC_50_ of 24.5 μM [17].

### 5.10. Antioxidant

The antioxidant activities of helioscopinolide A (**1**) inhibited COX 2 and iNOS mRNA [17].

### 5.11. Anti-Allergic Activity

Helioscopinolide A (**1**), helioscopinolide C **(2**), and helioscopinolide I **(3**) exhibited significant anti-allergic effects towards antigen-induced β-hexosaminidase release with the IC_50_ of 26.5, 37.0 and 29.3 µM, respectively [33]. *Ent*-16-kauren-3*β*,15*β*,18-triol (**60**), *ent*-3-oxo-16-kauren-15β,18-diol (**61**), *ent*-16-kaurene-3β,15β-diol (**62**) and abbeokutone (**63**) revealed noticeable anti-allergic effect against antigen-induced β hexosaminidase release with the IC_50_ of 22.5, 22.9 28,7 and 42.1 µM, respectively [36].

### 5.12. Antileishmanial Activity

The aqueous extract of *S. zanzibariensis* leaves revealed anti-leishmanial activity when tested on Leishmania major promastigotes with a mortality percentage of 40.5 ± 1.99%. The methanol extract revealed good anti-leishmanial activity on Leishmania major amastigotes with a mortality percentage of 28.0 ± 2.11%. Nitrogen production macrophages infected with amastigotes of Leishmania major treated with the *S. zanzibariensis* aqueous and methanol extracts showed significant Nitric oxide concentrations at 4.0 ± 0.56 and 6.6 ± 0.63 µg·mL^−1^ at 1000 µg·mL^−1^ [74]. The ethanol extract of *S. procera* revealed good antileishmanial activity with the IC_50_ of ≤10 µg·mL^−1^ [76]. Gelomulide A (**6**) and G (**12**) displayed significant antileishmanial activity with the IC_50_ below 20 µg·mL^−1^ [47].

### 5.13. Antiplasmodial Activity

Methanol extract of *S. zanzibariensis* exhibited a good antiplasmodial effect against *Plasmodium falciparum* strains with the IC_50_ of 4.66 ± 0.22 µg·mL^−1^ chloroquine-resistant (W2) and 1.82 ± 0.07 µg·mL^−1^ chloroquine-sensitive (D6) [77]. Omulokoli et al. stated that *S. zanzibariensis* leaf extract revealed significant antiplasmodial activity with the IC_50_ of 1.5 µg·mL^−1^ against *Plasmodium falciparum* chloroquine-resistant (ENT36) and chloroquine-sensitive (K67) [77].

**Table 2:** Pharmacological activities of the *Suregada* genus.

Plant Species	Plant Part	Extract	Activity	Effect	References
*S. aequorea*	Unspecified	Dichloromethane	Anticancer	Exhibited activity against different human cancer cells with the IC_50_ of <20 µg·mL^−1^.	[35]
*S. angustifolia*	Stem bark	Methanol	Antibacterial	Revealed zone inhibition of *S. aureus* (32 mm), Enterobacter aerogenes (26 mm), *E. coli* (26 mm), and *P. vulgaris* (25 mm) at 20 mg·mL^−1^.	[9]
		Chloroform		Showed zone inhibition of *A. hydrophila* (40 mm), *K. pneumonia* (37 mm), *S. Aureus* (32 mm), and *E. coli* (40 mm) at 20 mg·mL^−1^.	
		Hexane		Exhibited zone inhibition of *A. hydrophila* (29 mm), *K. pneumonia* (29 mm), *P. vulgaris* (25 mm), *V. vulnificus* (25 mm), *V. parahaemolyticus* (25 mm), *V. cholera* (25 mm) at 20 mg·mL^−1^.	
*S. glomerulata*	Leaves	Water	Antidiabetic	Potent inhibition against α-glucosidase with the IC_50_ of 2.29 µg·mL^−1^.	[49]
*S. multiflora*	Roots	Methanol: Ethyl acetate (1:9)	Insecticidal activity	Revealed 100% mortality rate of *Tribolium castaneum*	[61]
	Leaves			Showed a 40% mortality rate of *Tribolium castaneum*	
			Antibacterial and Antimicrobial	Revealed mild activity against *E. coli*, *Sh. Flexneri*, *B. subtilis*, *S. aureus*, and *P. aeruginosa* with the of zone inhibition range of 5 ± 0.10 to 6 ± 0.13 mm.	[7]
	Roots			Exhibited significant activity against *E. coli* with zone inhibition of 13 ± 0.01 mm and MIC of 0.625 mg·mL^−1^.	[61]
	Stem bark	Unspecified	Inflammatory activity	Exhibited NO inhibitory effect with the IC_50_ of 8.6 µg·mL^−1^.	[17]
*S. procera*	Unspecified	Ethanol	Anti-leishmanial activity	Exhibited strong activity, IC_50_ value ≤ 10 µg·mL^−1^	[76]
*S. zanzibariensis*	Leaves	Methanol	Anti-plasmodial activity	Revealed good anti-plasmodial properties against *Plasmodium falciparum* strains (W2 and D6) with IC_50_ of 4.66 ± 0.22 µg·mL^−1^ and 1.82 ± 0.07 µg·mL^−1^.	[74]
				Showed anti-plasmodial activity (1.5 µg·mL^−1^) against *Plasmodium falciparum* ENT36 and K67 with the IC_50_ of 1.5 µg·mL^−1^.	[71]
		Aqueous extract	Anti-leishmanial activity	Revealed anti-leishmanial activity on Leishmanial major promastigotes and amastigotes with a mortality percentage of 40.5 ± 1.99%.	[74]
		Methanol extracts		Possessed good anti-leishmanial activity on Leishmania major amastigotes with a mortality percentage of 28.0 ± 2.11%.	
				Revealed substantial differences in the production of NO by macrophages infected with Leishmania major amastigotes (6.6 ± 0.63 μM).	
		Aqueous extracts		Showed a significant difference in the production of nitric oxide by macrophages infected with Leishmania major amastigotes (4.0 ± 0.56 μM).	
			In vitro cytotoxicity	Low toxicity was observed against HELF cells with a cytotoxic concentration of 50% (CC_50_) value > 20 µg·mL^−1^.	
		Methanol extracts		Showed low toxicity against HELF cells with the CC_50_ > 20 µg·mL^−1^.	[78]
	Stem bark	Dichloromethane/methanol	Anticancer	Showed potent anticancer activity against TK10 with the TGI and GI_50_ of 0.60 µg·mL^−1^ and 0.26 µg·mL^−1^.	[50]
				Revealed anticancer activity against UACC62 with the TGI and GI_50_ 0.54 µg·mL^−1^ for and 0.25 µg·mL^−1^.	[50]
				Showed anticancer activity against MCF7 with the TGI and GI_50_ 5.27 µg·mL^−1^ and 0.81 µg·mL^−1^.	

## 6. Comparison of Ethnomedicinal Uses with the Pharmacological Uses

Several plant species of the *Suregada* genus are utilized by locals in traditional medicine against various ailments such as headaches and colds, dysentery, malaria, placenta apposition, epilepsy, skin diseases, worms, weakness, blood vomiting, piles, toothache, eczema, venereal diseases, pyrexia, lymphatic disorders, hepatitis, fungal infection, leprosy, fever, poisonous effects, stomach disorder, squint eye, gum disease, asthma, dysentery, vaginal candidiasis, abdominal pains, wound healing and ankylostomiasis and also as purgative, an astringent and against snakebite. Pharmacological studies were conducted on various species of this genus, such as anti-inflammatory, anticancer, antiviral, antidiabetic, antimicrobial, antileishmanial, antiplasmodial, cytotoxic, antioxidant, and insecticidal activity. A comparison of traditional medicinal uses of genus *Suregada* with the pharmacological studies is described as follows:

- The methanol and hexane extracts of *S. anguistifolia*, showed a maximum antibacterial effect against *E. coli*, *A. hydrophila*, and *K. pneumonia*. A similar activity was observed in *S. aureus* in chloroform and methanol extracts of *S. anguistifolia* stem bark. *Staphylococcus aureus* bacteria cause toothache and skin infections [9]. The pharmacological results from this plant species support the claims of traditional medicinal uses of *S. anguistifolia*, where Indian people in Kanis utilize it to treat skin infections and toothache [9].
- The wood of *S. multiflora* was reported to treat pyrexia, eczema, and venereal diseases, and the roots are utilized to treat lymphatic disorders and skin infections [17]. In Thailand, *S. multiflora* is utilized to treat skin diseases and inflammation [17]. Various solvent extracts from *S. multliflora* were screened for antibacterial and antimicrobial activity and exhibited effects against *B. subtilis*, *P. aeruginosa*, *Shi. flexineri*, *S. aureus*, and *E. coli* [62]. *S. aureus* is responsible for skin infections, gum diseases, eczema, and pyrexia (fever). The bacteria *P. aeruginosa* is responsible for lymphatic disorders, which can include swelling. *E. coli* and *shi. flexineri* responsible for pyrexia (fever) [76]. Helioscopinolide A (**1**) and epifriedelinol (**75**) isolated from *S. multiflora* revealed antibacterial activity that further substantiates the traditional uses claims of *S. multiflora*. Epifriedelinol (**75**) exhibited the highest zone inhibition against *S. aureus. S. aureus* is the bacteria responsible for skin infection, eczema, and gum diseases [57,59,71]. Suregadolide (**4**) isolated from *S. multiflora* showed antifungal activity, which confirms the claims of the traditional uses of the plant being utilized for treating fungal infections and skin disease [34].
- In some regions, the granule products of this species can be prepared, which acts as a powerful organic herbicide [19]. When tested for insecticidal activity, the (1:9) methanol: ethyl acetate root extract of *S. multiflora* revealed a mortality rate of 100% for *Tribolium castaneum* at 50 mg·mL^−1^ dose in 12 h [61]. Furthermore, the dichloromethane extract of *S. multiflora* stem showed partial antibacterial activity with zone inhibition of 3 mm against *X. campestris* [55]. *S. multiflora* revealed an antibacterial activity against *X. campestris*, which is responsible for plant diseases and insecticidal activity, which confirms the claims that the granules of *S. multiflora* act as organic herbicide [55,78].
- In Thailand, *S. multiflora* is used to treat inflammation [18]. The stem bark of *S. multiflora* showed anti-inflammatory properties against lipopolysaccharide (LPS)-induced nitric oxide (NO) and prostaglandin E (2) (PGE(2)) releases in RAW264.7 cells, and the anti-inflammatory mechanism on mRNA expression was carried out [17].
- A mixture of *S. multiflorum* is mixed with other herbs it is used as an anticancer recipe [17]. Helioscopinolide A (**5**) and gelomulide E (**12**) isolated from *S. multiflora* showed anticancer activity against various types of cancer, namely, leukemia (CCRF CEM), leukemia (SR), leukemia (K-562), breast (MD-MB-435), and colon (HTC-15) which supports the claims of *S. multiflora* being used traditionally in the anticancer recipe.
- *S. zanzibariensis* leaves are utilized to treat malaria. The leaf extract of *S. zanzibariensis* revealed high anti-plasmodial activity with the IC_50_ value of1.5 µg·mL^−1^) against *Plasmodium falciparum* K67 and ENT36 [77].
- Giriama and Duruma people use a root decoction to treat body swelling [28]. Tanzanian people used the stem bark and root extract of *S. zanzibariensis* to treat ankylostomiasis caused by parasitic hookworms [25]. Nitrogen production macrophages infected with amastigotes of Leishmania major treated with the methanol and aqueous extracts of *S. zanzibariensis* showed significant Nitric oxide concentration at 4.0 ± 0.56 and 6.6 ± 0.63 µg·mL^−1^ at 1000 µg·mL^−1^ [74]. The potent activity of this species against Leishmania, a parasitic disease, supports the claim that *S. zanzibariensis* is used to treat ankylostomiasis and body swelling. Simiarenol (**81**) isolated from *S. zanzibariensis* exhibited noticeable antinociceptive properties with the ID_50_ of 18.87 (14.6–24.4) mmol·kg^−1^ [71,72]. The activity of Simiarenol validates the claims of *S. zanziberiensis* being traditionally used for chest and abdominal pains.

## 7. Conclusions

The objective of this review was to outline the previous findings on the genus *Suregada* focusing on phytochemicals, pharmacological activities, and medicinal uses of the extracts and phytochemicals isolated from *Suregada* the species. The results showed that the genus *Suregada* is important due to its traditional medicinal benefits. This genus contains compounds that could be further explored for treating various ailments.

**Figure 13:**
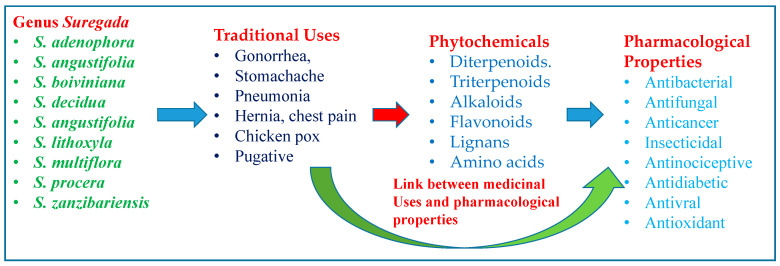
Summary of the ethnomedicinal, phytochemistry, and pharmacological properties of *Suregada* species.

The *Suregada* species are traditionally utilized in treating gum and hepatic diseases, mixed with other herbs, and used as an anticancer recipe to treat pyrexia, eczema, venereal diseases, lymphatic disorder, skin infection, skin infections, toothache, ankylostomiasis, gonorrhea, and stomach-ache. Pharmacological studies revealed that the compounds in *the Suregada* species exhibit diverse biological activity, including antibacterial, antimicrobial, antiplasmodial, anticancer, and antiviral as in Figure 13.

A significant number of investigations have been conducted on *S. multiflora* and *S. zanzibariensis*; nevertheless, other species have not been widely investigated. *Suregada* species, mainly. *S. multiflora* and *S. zanzibariensis* are utilized traditionally in the treatment of various illnesses. *S. zanzibariensis* root extract is drunk to treat gonorrhea, stomachache, pneumonia, hernia, chest pains, chicken pox, and as a purgative. There is a need for further evaluation of this species since the relationship between traditional and pharmacological uses is not clearly shown. The antimicrobial studies for *S. zanzibariensis* should be performed to substantiate the mentioned claims. Furthermore, *S. zanzibariensis* is traditionally used to treat vaginal candidiasis, which may be yeast or fungal infection; conducting antifungal candida studies to substantiate the claims is needed. *Suregada multiflorum* is traditionally used to treat venereal diseases and hepatitis. The antiviral activity of these plants and their phytochemicals should be studied. Future pharmacological and phytochemical investigations of African *Suregada* species should focus on other traditionally used and accepted species, such as *S. procera*, which is used to treat hemorrhoids and gonorrhea. The other six African species are accepted species, but their traditional uses are not known, namely, *S. africana*, *S. croizatiana*, *S. gossweileri*, *S. ivorense*, *S. lithoxylia*, and *S. occidentale*. Other *Suregada* species (*S. decidua*, *S. boiviniana*, and *S. adenophora*) have known traditional uses but have not been investigated for their pharmacological uses and phytochemistry.

The presence of biologically active tested phytochemicals in *Suregada* species could afford an important basis in the discovery of drugs. Moreover, most of the activities investigated so far are in vitro testing, and no in vivo screenings were performed. The biochemical interaction through which the extracts and isolated compounds of *Suregada* produce its pharmacological effects of displaying promising activities should be further investigated.

## Data Availability

Data sharing is not applicable.

## Lists of Abbreviations

A2780	Human ovarian cancer cell line
Aca-109	Human oesophageal carcinoma cell line
ATP	Adenosine triphosphate
Bax	pro-apoptosis gene
B16F10	Mouse melanoma cell line
Bcl-2	Anti-apoptosis gene
Bel 7402	Human liver cancer cell
BGC 823	Human stomach cancer cell
BT474	Breast cancer cell line
CC_50_	50% cytotoxic concentration
CCRF-CEM	Human leukemic lymphoblast cell line
COX-2	Cyclooxygenase-2
CRC	Colorectal cancer cell line
D6	chloroquine-sensitive
DCM	Dichloromethane
DNA	Deoxyribonucleic acid
DRC	Democratic Republic of Congo
ENT36	chloroquine-resistant
ERK	Extracellular signal-regulated kinase
GAP3	Glycosylation-associated protein
GI_50_	(50% growth inhibition)
Glut1, Glut3 and Glut4	Glucose transporter genes
K-562	Human chronic myeloid leukemia cell line
K67	chloroquine-resistant
H37Ra	Mycobacterium tuberculosis
HCT-8	Human colon cancer cell line
HTC-15	Human colon cancer cell line
HeLa	Human cervical cancer cell line
HELF	Human embryonic lung fibroblast
HepG2	(hepatocellular carcinoma) Liver cancer cell line
Hk2	Hexokinase 2
HIV	Human Immunodeficiency Virus
HSV	Herpes simplex virus
HT29	Human colon cancer cell line
IC_12_	Minimum concentration that gives an inhibition zone of 12 mm diameter
IC_50_	Half-maximal inhibitory concentration
iNOS	Inducible nitric oxide synthase
Ldha	Lactate dehydrogenase-A
LPS	Lipopolysaccharide
MCF7	human breast cancer cell line
MDA-MB-231	Epithelial, human breast cancer cell line
MDA-MB435	Epithelial, human breast cancer cell line
MIC	Minimal inhibitory concentration
mRNA	Messenger Ribonucleic acid
NCI	National Cancer Institute
NCI-H460	Non-small cell lung cancer
NCI-H490	Lung cancer cell line
NO	Nitric oxide
NRF	National Research Foundation
PGE(2)	Prostaglandin E(2)
RAW 264.7	Mouse macrophage cell line
ROS	Reactive oxygen species
SKBR3	Breast cancer cell line
SR	Spontaneous remission leukemia cell line
SW620	Human colon cancer cell line
TGI	Total growth inhibition
TK10	Renal cancer
UACC62	Melanoma cell line
U937	Human leukemic cell line
*W*2	chloroquine-resistant
WHO	World Health Organisation
µg/mL	Micrograms per milliliter
µmol/L	Micromole per litre
µg/spot	Micrograms per spot
